# The psychological burden of waiting for procedures and patient‐centred strategies that could support the mental health of wait‐listed patients and caregivers during the COVID‐19 pandemic: A scoping review

**DOI:** 10.1111/hex.13241

**Published:** 2021-03-26

**Authors:** Anna R. Gagliardi, Cindy Y. Y. Yip, Jonathan Irish, Frances C. Wright, Barry Rubin, Heather Ross, Robin Green, Susan Abbey, Mary Pat McAndrews, Donna E. Stewart

**Affiliations:** ^1^ Toronto General Hospital Research Institute University Health Network Toronto Canada; ^2^ University of British Columbia Vancouver Canada; ^3^ Surgical Oncology Program/Access to Care‐Surgery Ontario Health‐Cancer Care Ontario Toronto Canada; ^4^ Department of Surgery Sunnybrook Health Sciences Centre Toronto Canada; ^5^ Peter Munk Cardiac Centre University Health Network Toronto Canada; ^6^ Ted Rogers Centre of Excellence in Heart Function University Health Network Toronto Canada; ^7^ Toronto Rehabilitation Institute University Health Network Toronto Canada; ^8^ Medical Psychiatry & Psychosocial Oncology University Health Network Toronto Canada; ^9^ Krembil Research Institute University Health Network Toronto Canada; ^10^ University Health Network Centre for Mental Health University of Toronto Toronto Canada

**Keywords:** anxiety, depression, implementation science, mental health, patient‐centred care, quality improvement, quality of life, review, waiting lists

## Abstract

**Background:**

Waiting for procedures delayed by COVID‐19 may cause anxiety and related adverse consequences.

**Objective:**

To synthesize research on the mental health impact of waiting and patient‐centred mitigation strategies that could be applied in the COVID‐19 context.

**Methods:**

Using a scoping review approach, we searched 9 databases for studies on waiting lists and mental health and reported study characteristics, impacts and intervention attributes and outcomes.

**Results:**

We included 51 studies that focussed on organ transplant (60.8%), surgery (21.6%) or cancer management (13.7%). Most patients and caregivers reported anxiety, depression and poor quality of life, which deteriorated with increasing wait time. The impact of waiting on mental health was greater among women and new immigrants, and those of younger age, lower socio‐economic status, or with less‐positive coping ability. Six studies evaluated educational strategies to develop coping skills: 2 reduced depression (2 did not), 1 reduced anxiety (2 did not) and 2 improved quality of life (2 did not). In contrast, patients desired acknowledgement of concerns, peer support, and periodic communication about wait‐list position, prioritization criteria and anticipated procedure date.

**Conclusions:**

Findings revealed patient‐centred strategies to alleviate the mental health impact of waiting for procedures. Ongoing research should explore how to optimize the impact of those strategies for diverse patients and caregivers, particularly in the COVID‐19 context.

**Patient or Public Contribution:**

Six patients and four caregivers waiting for COVID‐19‐delayed procedures helped to establish eligibility criteria, plan data extraction and review a draft and final report.

## 1 INTRODUCTION

Hospitals worldwide have focussed resources on COVID‐19, and as a result, many patients with debilitating or life‐threatening illness are waiting for procedures (e.g. tests, surgery, other therapy).^1^, ^2^, ^3^ The problem is widespread, as an estimated 28,404,603 operations in 190 countries may have been cancelled or delayed during 12 weeks of peak COVID‐19 disruption.^4^ Doing so has created lengthy procedure backlogs that will not be resolved for many weeks or months. Assuming immediate return to normal service, modelling showed that it would require 84 weeks to clear the surgical backlog, and for time‐sensitive surgeries only (cardiac, cancer, vascular and transplant), it would require approximately 14 weeks to clear the backlog provided that all surgical resources were dedicated to time‐sensitive surgeries.^5^ Given the ongoing nature of the current pandemic, this problem may not be resolved for quite some time, leaving millions of patients worldwide waiting for essential health care.

Delayed procedures may be associated with disease progression and mortality.^6^, ^7^ Moreover, wait‐listed patients may experience anxiety, which can manifest physically (e.g. heart palpitations, gastrointestinal symptoms), prompt or worsen other distinct aspects of mental health (e.g. depression, substance use) and escalate if untreated.^8^ Mental health may be worse in those waiting for time‐sensitive procedures (e.g. cardiac or cancer surgery) among whom anxiety and depression are common and linked to elevated mortality, compared with procedures that impact quality of life but are less time‐sensitive (e.g. cataracts, joint replacement).^9^, ^10^ Already‐strained health systems may face added future pressure to manage mental health needs emerging from the pandemic.^11^ Thus, patient‐centred strategies are needed to prevent or alleviate the mental health impact on patients waiting for procedures. Patient‐centred care is widely advocated as a fundamental component of high‐quality care because it leads to many positive outcomes for patients, family and health‐care professionals across health‐care settings and jurisdictions.^12^, ^13^

Prior research on the psychological impact of infectious outbreaks (e.g. SARS, Ebola) did not include assessment of wait‐listed patients.^14^ Recent pandemic research focussed on only the logistics of managing wait lists.^15^ Similarly, surgical policy across jurisdictions focussed on prioritizing procedures, screening patients and protective equipment requirements, with no specific guidance on patient‐centred communication about delays.^16^ There is no recent or thorough synthesis on mental health and wait lists, a long‐studied health‐care issue and untapped source of knowledge to address this gap.^17^ We aimed to synthesize published research on patient‐centred strategies to support mental health among patients (and caregivers) waiting for procedures and identify knowledge that could potentially be applied in the COVID‐19 context. Our objectives were to describe the following: (a) Mental health impact of waiting on diverse patient groups; (b) Determinants of the mental health impact of waiting; and (c) Attributes and effectiveness of strategies to support mental health among wait‐listed patients.

## 2 METHODS

### 2.1 Approach

We conducted a scoping review comprised of six steps: scoping, searching, screening, data extraction, data analysis and collaborator interpretation of findings; and complied with standard methods,^18^, ^19^ and a reporting checklist specific to scoping reviews.^20^ Similar in rigour to a systematic review, we chose a scoping review because it includes a range of study designs and outcomes to reveal existing knowledge and identify issues requiring further primary study.^18^, ^19^, ^20^, ^21^ As this research was funded by a COVID‐19 opportunity that required results in one month, we also employed a rapid review approach, characterized by single language (English), short time frame (last 10 years), exclusion of grey literature and non‐duplicate screening/data extraction.^22^ We did not require research ethics board approval as data were publicly available, and we did not register a protocol. The research team, collaborators and patient/family research partners informed the study at four points: established eligibility criteria, reviewed a preliminary summary of extracted data, reviewed a draft report and reviewed the final report.

### 2.2 Scoping

We conducted an exploratory search in MEDLINE using Medical Subject Headings: waiting lists AND anxiety or psychological distress or stress, psychological. By reviewing examples of relevant studies, we generated eligibility criteria based on the PICO (participants, issue, comparisons, outcomes) framework and planned a more elaborate search strategy.

### 2.3 Eligibility

Table 1 specifies inclusion criteria. In brief, we included studies that assessed the impact of waiting on patients with any of the distinct aspects of mental health (e.g. anxiety, stress, distress, depression) or their families; determinants of the impact of waiting on mental health, and the effectiveness of strategies to support mental health while waiting. Study design included qualitative, quantitative or multiple/mixed methods. While waiting for health‐care services may exacerbate symptoms among those with mental health conditions, which is an important health‐care concern, we excluded studies that measured mental health not related to waiting for procedures so that findings unambiguously reflected the impact of waiting rather than an underlying health‐care issue. Studies referring to usual care as ‘wait‐list controls’, assessing anxiety directly prior to appointments or procedures, based on waiting for results of procedures, or involving patients who chose watching waiting/active surveillance were not eligible, nor were publications in the form of protocols, abstracts, editorials or letters to the editor.

**TABLE 1:** Study inclusion criteria

Category	Criteria
Participants	Patients and/or caregivers of any socio‐demographic characteristics waiting any length of time to see a specialist for diagnosis or to undergo a medical procedure, where ‘procedure’ referred to tests or therapy performed in hospitals or outpatient clinics by any clinicians, therapists or technicians.
Issue	Impact of waiting on any aspect of mental health including but not limited to: anxiety, stress, distress, depression or psychological impact, etc
Comparisons	Exploring or describing the impact of waiting on mental health, determinants of the impact of waiting on mental health, and the effectiveness of strategies to support mental health while waiting. Determinants referred to characteristics or behaviours of patients, caregivers or health‐care professionals or characteristics of health‐care systems. Strategies referred to approaches, programmes, interventions or tools implemented to support mental health
Study design	Qualitative, quantitative or multiple/mixed methods. Reviews were not eligible, but we screened review references for eligible primary studies
Outcomes	Any mental health impact of waiting Related somatic, lifestyle or other behavioural sequelae Determinants of mental health among patients or caregivers Effectiveness (benefits, harms) of strategies for patients, caregivers, health‐care professionals or the health‐care system

### 2.4 Searching and screening

ARG, who has medical librarian training, developed a search strategy (Table S1) that complied with the Peer Review of Electronic Search Strategy reporting guidelines.^23^ We searched MEDLINE, EMBASE, CINAHL, SCOPUS, Allied and Complementary Medicine, PsychInfo, Sociological Abstracts, the Cochrane Library and Joanna Briggs Institute Database of Systematic Reviews from 1 January 2010 to 8 July 2020. ARG and a research associate (RA) independently screened the same 50 titles and abstracts and disagreed on the eligibility of one item, leading to a clarification in eligibility criteria that quality of life assessment must pertain to the impact of wait‐listing and not solely on physiological factors. ARG screened remaining titles and abstracts, and retrieved and screened full‐text articles concurrent with data extraction.

### 2.5 Data extraction and analysis

We extracted data on study attributes (author, publication year, country, goal, disease, wait‐listed procedure, research design, participants), mental health impact of waiting (instruments used, results), determinants of the impact of waiting on mental health (those reported by studies), and strategies to support mental health (design, effectiveness). We described strategies using the Workgroup for Intervention Development and Evaluation Research reporting framework (content, format, delivery, timing, personnel).^24^ ARG extracted and tabulated data, and used summary statistics, tables and text to report study characteristics and results. We did not assess methodological quality of included studies as this is not required of scoping or rapid reviews.^18^, ^19^, ^20^, ^21^, ^22^ We could not undertake further statistical analyses to combine outcomes across studies as they varied widely by disease, procedure, study design and outcomes.

## 3 RESULTS

### 3.1 Search results

**FIGURE 1:**
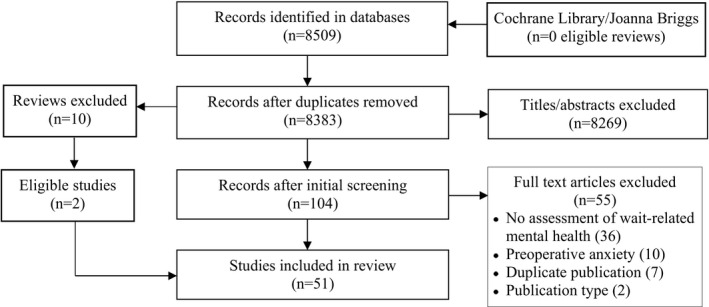
PRISMA diagram. PRISMA flow diagram of studies identified, screened and included

We identified 8509 primary studies, 8383 were unique, and 8269 were excluded based on title/abstract screening. Among 104 full‐text articles screened, we excluded 55 studies that did not assess mental health related to waiting (36), focussed on pre‐procedure anxiety not related to waiting (10) or were a duplicate (7) or ineligible type of publication (2). Among 10 excluded reviews, we identified 2 unique eligible studies. We included 51 studies in this review (Figure 1). Table S2 reports extracted data.^25^, ^26^, ^27^, ^28^, ^29^, ^30^, ^31^, ^32^, ^33^, ^34^, ^35^, ^36^, ^37^, ^38^, ^39^, ^40^, ^41^, ^42^, ^43^, ^44^, ^45^, ^46^, ^47^, ^48^, ^49^, ^50^, ^51^, ^52^, ^53^, ^54^, ^55^, ^56^, ^57^, ^58^, ^59^, ^60^, ^61^, ^62^, ^63^, ^64^, ^65^, ^66^, ^67^, ^68^, ^69^, ^70^, ^71^, ^72^, ^73^, ^74^, ^75^, ^76^

### 3.2 Study characteristics

Studies were conducted in 19 countries and published from 2010 to 2019. Common research designs were surveys (29, 56.9%) and qualitative interviews or focus groups (12, 23.5%). Commonly used instruments were the Short Form Health Survey (7, 13.7%), Beck Depression Inventory (6, 11.8%), State Trait Anxiety Scale (6, 11.8%) and the Hospital Anxiety and Depression Scale (6, 11.8%). Most studies focussed on transplant surgery (31, 60.8%) including 11 kidney, 11 liver, 4 solid organ, 3 heart and 2 lung transplant studies. Other studies concerned surgery (11, 21.6%) including 3 orthopedic, 3 bariatric, 3 mixed, 1 sinus and 1 thyroid surgery; and cancer management (7, 13.7%) including 3 mixed, 2 gastrointestinal, 1 breast and 1 colorectal. One (2.0%) study pertained to chronic pain and 1 (2.0%) to specialist visits. Patients were participants in most studies (43, 84.3%). Fewer studies included both patients and caregivers (6, 11.8%) or caregivers only (2, 3.9%). Among studies involving caregivers, the proportion of women caregivers ranged from 74% to 83%. Twenty‐four (47.1%) studies reporting participant waiting time but using variable measures. For example, mean waiting time varied from 6.2 days to 6.0 years and median waiting time varied from 286 days to 60 months.

### 3.3 Impact of waiting on mental health

Of the 31 (60.8%) studies that assessed the mental health impact of waiting, 18 (58.1%) employed quantitative methods. Four (22.2%) studies demonstrated the emotional toll of waiting on caregivers. In one study, many caregivers had depression (75.4%), difficulty concentrating (75.0%) and insomnia (44%) and had ceased employment (59.0%) or social activities (41.0%).^76^ Caregiver anxiety was greater than patients upon wait‐listing (F = 7.52, *P* =.008) and at 6 months (F = 11.31, *P* = .002) and increased over time, but scores did not differ for depression, which remained stable over time for both groups.^55^ Another study similarly found that anxiety was greater among caregivers (mean 10.80, SD 5.07, *P* = .001) but depression was similar (mean 6.65, SD 4.07, *P* = .820) to patients.^73^ Patients whose caregivers had lower anxiety or depression were more likely to report positive coping through social support (*P* = .007), emotional control (*P* = .030) and active fighting (*P* = .032).^61^

Among 14 (77.8%) patient‐only quantitative studies, most assessed anxiety and/or depression (9, 64.3%) followed by quality of life (6, 42.9%). Eleven to 98% of patients reported clinical anxiety or depression (median 64.0%).^28^, ^46^, ^48^, ^56^, ^63^, ^67^ Other studies reported mean anxiety: 14.7 (SD 8.6), 23.1 (SD 5.5), 8.6 (SD 4.6) or mean depression: 14.7 (SD 8.6), 11.8 (SD 3.3).^43^, ^48^, ^58^ Three studies showed that anxiety (*P* = .006; F = 2.06, range 3 to 256, *P* = .08) and depression (F = 6.35, range 3 to 256, *P* = .0004) increased over time and decreased after surgery (*P* = .0157).^56^, ^58^, ^72^ In one study, 19% of wait‐listed patients reported poor quality of life.^39^ In another study, the mental health component was similarly impacted among women undergoing pelvic prolapse surgery, or hip or knee replacement (41.5 vs 44.6, *P* = .09).^38^ Three studies showed that social function (mean 75.9, SD 22.7; r = 0.319, *P* = .0012; *P* = .05) and mental health (mean 46.2, SD 10.6; r = 0.3832, *P* = .001; *P* = .01) quality of life components were particularly affected.^57^, ^67^, ^69^ One study showed that quality of life deteriorated over time (mean change −0.04, 95% CI −0.08 to −0.01, *P* = .02).^68^

Thirteen (41.9%) qualitative studies involved a mean of 21.5 participants (median 16, range 6 to 60) who were waiting for cancer procedures, organ transplant, or surgery (bariatric, mixed, sinus). Table 2 summarizes themes. Participants mentioned *depression* and/or *anxiety*, noting they increased over time. *Uncertainty* pertained to length of the wait, and whether health was deteriorating to such an extent that it might influence eligibility for the wait‐listed procedure or clinical outcomes of the procedure. *Restriction* referred to inability to perform physical functions due to immobility or pain, and inability to work or take part in social activities. *Life on hold* was in part due to restriction, but also being constantly on alert for a phone call, and unable to make future plans as a result of health limitations or uncertainty about timing of the procedure. *Coping strategies* varied across individuals and included acceptance, distraction, social support and constructive use of time. Participants said that it was exhausting to deal with the gamut of impacts that pervaded all aspects of their lives, referring to it as a ‘daily emotional roller‐coaster’ and an ‘immense struggle’. Exhaustion increased over time, reducing motivation to maintain a healthy lifestyle, and turning hope into despair. As a result, *trust in the health‐care system* eroded. Participants felt anger that they were not considered a priority and ‘at the mercy of the health‐care system’ and frustrated with the lack of information, and ambiguity and perceived inequity in prioritization. Even if waiting times could not be reduced, participants recommended three strategies that would alleviate the mental health impact of waiting and assure them they had not ‘fallen through the cracks’: (a) To be able to report the mental health impact of waiting to health‐care professionals and have them acknowledge and empathize with those concerns; (b) Periodic updates from health‐care professionals that included the reason for delay, position on the waiting list, prioritization criteria and an anticipated procedure date; and (c) Interventions to help them through the waiting period including peer support (support group, peer mentor), and health and mental health counselling.

**TABLE 2:** Themes about the impact of waiting on mental health identified in qualitative studies

Study	Theme (n,% of 13 studies)						
	Uncertainty about condition	Life on hold	Restricted activities	Depression	Anxiety	Coping	Trust in health‐care system
Burns 2017^33^	+		+			+	
Carr 2017^34^	+		+	+	+	+	
Sharman 2017^41^				+	+		+
Fung‐Zak Tsang 2016^43^	+			+	+	+	+
Jin Chong 2016^44^	+	+					+
Yngman‐Uhlin 2016^49^		+			+		+
Matthews 2015^50^							+
Anthony 2014^51^			+	+	+		
Brugger 2014^52^	+			+	+	+	
Gregory 2013^59^	+				+		+
Yelle 2013^60^		+		+			+
Moran 2011^70^	+	+		+			
Mulcahy 2010^74^				+			+
Total themes	7 (53.8)	4 (30.8)	3 (23.1)	8 (61.5)	7 (53.8)	4 (30.8)	8 (61.5)

We could not compare the impact of waiting by disease or procedure due to the limited number and variability of studies. Among 7 studies of cancer procedures (3 mixed, 2 gastrointestinal, 1 breast, 1 colorectal), waiting caused anxiety and depression and reduced quality of life across the range of waiting periods (e.g. specialist visit, diagnosis, decision, treatment), which was said to be frustrating, demoralizing and traumatizing, but alleviated by communication from physicians with good interpersonal skills.^37^, ^39^, ^40^, ^50^, ^66^, ^74^, ^75^ Among 11 studies of surgical procedures (3 mixed, 3 bariatric, 3 orthopedic, 1 sinus, 1 thyroid), waiting caused anxiety and depression and reduced quality of life due to restricted activities, concern about worsening health and uncertainty about procedure date.^34^, ^65^, ^68^

### 3.4 Determinants of the mental health impact of waiting

Sixteen (31.4%) studies assessed factors that influenced the mental health impact of waiting.

One study of caregivers found that caregiver burden scores were negatively associated with depression (b = 0.43, *P* < .001), and caregiver burden (b = 0.38, *P* < .001) and avoidant coping style (b = 0.17, *P* = .002) were negatively associated with anxiety.^62^ In one study involving both patients and caregivers, lung transplant patients had greater anxiety compared with heart transplant patients (*P* = .04); and lung cancer patients (*P* = .04) and patients < 50 years of age (*P* = .029) had greater coping ability.^30^ The same study found that 22.9% of caregivers had medium to high burden levels; determinants were not reported.

Table 3 summarizes determinants of the mental health impact of waiting in studies of patients (14, 87.5%). Of those, 2 studies found no association of age, sex or wait time with coping,^40^ or sex on depression (mean 21.31, SD 12.82, *P* = .06).^53^ In other studies, anxiety and depression were increased by negative coping style, being female, younger age, poor quality of life, neurosurgery vs other type of elective surgery, new immigrant, longer wait time, wait time perceived as too long, and low self‐estimated chance of having the procedure.^31^, ^37^, ^45^, ^47^, ^54^, ^64^, ^75^ Quality of life was reduced by older age and being female,^29^, ^53^ and improved in one study by being married, employed, on haemodialysis and Chinese (who represent the majority of Singaporeans vs Indigenous Malays).^57^ Satisfaction with care was reduced with lower health status scores, unemployment and being depressed.^65^ Hope was enhanced by perceived social support.^25^ Concern about waiting in an Australian study was higher among those of younger age, lower socio‐economic status and born elsewhere.^66^ Among the 45 studies that assessed impact and/or determinants of the impact of waiting on mental health, few (6, 13.3%) reported the race, ethnicity or culture of participants or assessed the influence of those factors on mental health while waiting (4, 8.9%).

**TABLE 3:** Determinants of the impact of waiting on mental health

Study	Dependent variables	Association of independent variables
Goktas 2019^25^	Hope	Perceived social support (r = 0.276, *P* = .001)
Lonning 2018^29^	Quality of life	Older age (*P* < .05)
Annema 2017^31^	Anxiety, depression	Emotional rather than task‐oriented coping style (*P* < .001 anxiety, *P* < .01 depression)
Hayes 2017^37^	Anxiety	Increasing wait time (B = 0.65, SE = 0.24, *P* = .008) Caucasian female (B = 6.38, SE = 2.30, *P* = .006)
Nagao 2017^40^	Coping style	*Not significant: age, female, waiting time*
Khatib 2016^45^	Anxiety, depression	Female (*P* = .025) Younger age (*P* < .001) Lower quality of life (*P* < .001)
Sutherland 2016^47^	Depression	Neurosurgery vs other types of elective surgery (*P* < .01) Age < 60 (*P* < .01)
dos Santos Cunha 2014^53^	Depression	*Not significant: female (mean 21.31,* SD *12.82, P = .06)*
	Quality of life	Female (emotional health *P* = .04; mental health *P* = .02)
Harrington 2014^54^	Anxiety	Female (OR 0.74, *P* < .05) Age 30 to 59 (OR 1.49, *P* < .05) New immigrant < 10 years (OR 1.95, *P* < .05) Wait time (OR 2.78, *P* < .001) Wait viewed as too long (OR 11.3, *P* < .001)
Chin Ong 2013^57^	Quality of life	Chinese (physical B=−2.68; mental B=−2.62) Married (physical B=−0.97; mental B=−4.35) Employed (physical B=−3.62; mental B=−2.97) On haemodialysis (physical B=−0.33; mental B = 0.78) All *P* < .05
Kam‐Tao Li 2012^64^	Happiness score given wait time	Low self‐estimated chance of procedure (*P* < .0001)
Padwal 2012^65^	Satisfaction with care given wait time	Lower health status scores (0.42, *P* = .03) Unemployed (13.7, *P* = .01) Being depressed (10.3, *P* = .003)
Paul 2012^66^	Concern about waiting	Lower socio‐economic status Born outside Australia Younger age
Parker 2010^75^	Anxiety	Coping styles of denial, disengagement, venting and self‐blame (R^2^ 0.527 to 0.563 for different components) Female (R^2^ 0.121) Increasing wait time (R^2^ 0.058)

### 3.5 Strategies to support mental health while waiting

Six (11.8%) studies evaluated strategies to support mental health (Table 4). All aimed to improve coping ability among patients waiting for organ transplant (5 studies) or chronic pain care (1 study). Findings were mixed regardless of group vs individualized therapy, number of sessions or session length or delivery mode (in‐person, telephone). Two before‐after studies evaluated in‐person group therapy. One study of 12 2.5‐hour sessions over 6 months involving 7 patients did not improve quality of life (2.8 before, 2.5 after, *P* = .28) but reduced depression (range 0 to 10 before, all scored 0 after).^26^, ^27^ The second study of 2‐hour sessions for 8 weeks involving 41 patients reduced both anxiety (13.0 ± 1.23 vs 7.73 ± 0.85, <0.001) and depression (14.23 ± 1.45 vs 7.73 ± 0.95, *P* < .0001).^35^ Two randomized controlled trials evaluated in‐person group therapy. One study of 3 1‐hour in‐person group sessions and 6 1.5‐hour group teleconferences involving 27 intervention group patients improved quality of life (6.2 points, 95% CI 1.66 to 10.8, *P* = .01) but not anxiety (−1.88, 95% CI − 8.14 to 4.37, *P* = .55) or depression (2.81, 95% CI 0.02 to 5.60, *P* = .05) compared with the control group.^36^ The second trial of a single 3‐hour session plus a handout involving 66 intervention group patients did not improve quality of life, distress or pain acceptance compared with the control group.^42^ Two randomized controlled trials evaluated individualized therapy. One study of 6 30‐minute telephone sessions over 12 weeks for 56 patient‐caregiver dyads in the intervention group improved self‐efficacy among patients (mean difference 3.1, 95% CI −4.4. to 10.7) and caregivers (mean difference 4.8 points, 95% CI −1.4 to 11.0) but not uncertainty, coping, anxiety or depression, but there was no significant difference in outcomes between intervention and control groups.^32^ The second study involving weekly 50 minute in‐person therapy over 8 weeks for 22 intervention patients improved quality of life (mean 45.8, SD 13.1, *P* < .05; SF36: mean 46.1, SD 9.6, *P* < .05) and reduced distress (mean 20.7, SD 16.1, *P* < .05; HSC: mean 38.6, SD 8.3, *P* < .05) compared with the control group.^71^ Four studies reported on the race, ethnicity or culture of participants (majority were Caucasian) but did not assess the impact of these factors on intervention outcomes.^32^, ^36^, ^42^, ^71^

**TABLE 4:** Design of strategies to support mental health of wait‐listed patients and caregivers

Study	Goal (Research Design)	Intervention Design				
		Content	Format	Delivery	Timing	Personnel
Febrero 2019, 2018^26^, ^27^	Impact of group psychotherapy on quality of life and depression (liver transplant)	Before‐after study: Feelings, emotions and coping strategies	Group discussion	In‐person	12 sessions of 2.5 h each every 2 wk for 6 mo	Psychologist and a social worker led sessions who facilitated discussion of emotions and their meaning
Bailey 2017^32^	Impact of phone call for uncertainty self‐management versus education (liver transplant)	Randomized controlled trial: Intervention Coping skills training, based on cognitive‐behavioural principles, to help patients change illness‐related thoughts, emotions and behaviours; symptom management strategies, based on Uncertainty in Illness Theory, designed to provide information about symptoms and strategies to decrease their frequency and intensity Control Liver function, disease aetiologies, stages of liver disease, diagnosing liver disease, common treatments, transplantation and staying healthy while waiting for a transplant	Intervention Didactic, interactive Control Didactic, interactive	Intervention Telephone Control Telephone	Intervention 6 sessions of 30 min over 12 wk Control 6 sessions of 30 min over 12 wk	Intervention Trained nurse or social worker Control Trained nurse or social worker
Craig 2017^35^	Impact of coping skills group therapy on coping, anxiety and depression (kidney or liver transplant)	Before‐after study: 8 modules; designed around cognitive‐behavioural, narrative and mindfulness interventions to enhance patients’ repertoire of coping skills that would allow them to better manage the psychosocial demands associated with the pre‐transplant experience	Groups of 7 to 10	In‐person	2 h sessions weekly for 8 wk (16 h total)	Social workers authorized to provide psychosocial interventions
Gross 2017^36^	Impact of phone mindfulness‐based stress reduction on anxiety, depression, HRQoL (kidney transplant)	Randomized controlled trial: Intervention Standard mindfulness‐based curriculum: introductory workshop yoga poses; teacher‐led meditations and discussions during teleconferences; final workshop ‘day of mindfulness’ retreat Control Building interpersonal communications skills and accessing reliable information from the Internet	Intervention Didactic, interactive Control Didactic, interactive	Intervention In‐person, telephone Control In‐person, telephone	Intervention 8 sessions total: 3‐hour in‐person workshop weeks 1 and 8, and 1.5 h group teleconference weeks 2 to 7 Control 2 1.5 h workshops at beginning and end with 1 h weekly teleconferences in between	Intervention Certified mindfulness‐based teacher Control Experienced group facilitator
Burke 2016^42^	Impact of a single education session on distress, quality of life and pain acceptance (chronic pain)	Randomized controlled trial: Intervention Goal of the session was to inform and encourage a psychological shift from the often fruitless quest for pain cessation or control, to a stance of acceptance and life engagement in the face of pain. Topics included chronic pain processes, the clinical unit and what to expect from treatment, the role of psychological factors in pain and ways to manage pain (e.g. relaxation, mindfulness and challenging thinking), goal setting, sleep hygiene, self‐care, distraction/attention focus, exercise, activity pacing and medication Control ‐‐‐	Intervention Didactic, interactive Control ‐‐‐	Intervention In‐person plus print handouts to reinforce session information Control ‐‐‐	Intervention 1 3‐hour session Control ‐‐‐	Intervention Pain consultant physician, psychologist and physiotherapist Control ‐‐‐
Rodrigue 2011^71^	Impact of quality of life therapy or supportive care therapy on quality of life and distress (kidney transplant)	Randomized controlled trial: Quality of life Tailored to patient needs. Identify quality of life issues and causes of dissatisfaction, develop a strategy to change perceptions, attitudes or behaviour, identify and develop skills, and measures of improvement Supportive care Emotional and educational support to develop coping skills. Topics included: understanding the transplant process, understanding medications and their effects, coping with illness and transplantation, identifying and dealing with emotions, dealing with issues of death and dying, communicating with others, and navigating the health‐care system Control ‐‐‐	Quality of life Interactive Supportive care Interactive Control ‐‐‐	Quality of life In‐person, individual Supportive care In‐person, individual Control ‐‐‐	Quality of life 50 min once weekly for 8 wk over 2 mo (full dose ≥ 6 wk) Supportive care 50 min once weekly for 8 wk over 2 mo (full dose ≥ 6 wk) Control ‐‐‐	Quality of life Master's or PhD level social workers and psychologists with at least 2‐year experience in transplantation Supportive care Master's or PhD level social workers and psychologists with at least 2‐year experience in transplantation (different person from quality of life therapy) Control ‐‐‐

## 4 DISCUSSION

This synthesis revealed that most patients and caregivers waiting for procedures had anxiety or depression, which adversely affected quality of life and eroded trust in the health‐care system. The impact of waiting on mental health was greater among women and new immigrants, and those of younger age, lower socio‐economic status, or with less‐positive coping ability or longer wait times. Coping skills training through multiple in‐person or online classes over many months did not consistently reduce anxiety or depression, or improve quality of life. Instead, patients said that acknowledgement of the burden of waiting, peer support and periodic communication to update wait‐list status could alleviate the mental health impact of waiting.

A pre‐COVID‐19 review of psychological outcomes among people exposed to infectious outbreaks (e.g. SARS, Ebola) included health‐care professionals and the public, but not wait‐listed patients.^12^ Similarly, research on psychological distress in response to the current pandemic focussed on the general public's response to COVID‐19 or mental health problems faced by health‐care workers.^77^, ^78^ Other reviews of literature on wait lists synthesized and reported wait times for emergent care and elective surgery, assessed the validity of instruments used to measure quality of life among wait‐listed patients or evaluated strategies to reduce anxiety among patients in waiting rooms directly before undergoing procedures.^79^, ^80^, ^81^ In contracts, we synthesized research on the mental health impact of waiting for procedures, and on strategies to support mental health among wait‐listed patients and caregivers.

Our findings suggest several implications for policy and practice. Patients and caregivers waiting for procedures experience anxiety, depression and poor quality of life, which escalates over time and can lead to future strain on the healthcare system.^8^, ^11^ Given that COVID‐19 policies focus on wait‐list management,^15^, ^16^ it may take up to two years to clear pandemic wait lists or longer if return to normal service is further delayed,^3^ and there is a known association between anxiety or depression and adverse outcomes,^9^, ^10^ strategies are needed to alleviate the mental health impact of waiting among patients and caregivers waiting for procedures cancelled or delayed by COVID‐19. Dedicated resources may be needed by hospitals to enhance their capacity for automating personalized wait‐list communication to thousands of affected patients.^82^ Doing so may, in turn, alleviate strain on clinicians and their staff who are unable to predict when procedures will be scheduled yet must respond to phone calls from anxious patients or caregivers. Given that self‐directed tools alone such as an informational handout on coping strategies can improve self‐efficacy, positive lifestyle behaviour and symptom control,^83^, ^84^ professional societies could develop guidance pertaining to mental health support, disease‐specific charities could develop or facilitate the delivery of information or education to patients and caregivers, and both could advocate to policymakers for needed resources.

This study generated insight on options for patient‐centred strategies that may support mental health among wait‐listed patients and caregivers. The complex educational strategies tested by included studies did not consistently reduce anxiety or depression, or improve quality of life and may not be feasible to replicate outside the context of funded research. While negative coping style among patients was associated with greater anxiety and depression in both patients and caregivers, participants did not articulate the need for improved coping. Instead, they suggested three strategies. One, participants wanted health‐care professionals to acknowledge the impact of waiting on their mental health. Addressing emotions is a recognized component of person‐centred care and includes eliciting or listening to concerns, expressing empathy, acknowledging hearing and understanding concerns, validating concerns by noting they are normal or common, and offering strategies to manage emotions or referring individuals to helpful information or services.^85^, ^86^ Person‐centred care has been associated with increased knowledge, skill, quality of life and satisfaction with the health‐care system; and decreased stress and anxiety among patients and caregivers across primary, emergency, acute and intensive care settings.^87^, ^88^ A related concept is that of safety‐netting, where clinicians explicitly address uncertainty by providing advice on what to do and who to contact if symptoms should arise, and subsequently monitoring for symptoms and/or arranging follow‐up care.^89^ Two, participants said that peer support groups or peer mentoring could help them withstand the waiting period. Peer support can be delivered in a variety of ways and was both feasible and effective for a variety of conditions.^90^ In a meta‐analysis, peer support interventions for depression were found to be just as effective as cognitive behavioural therapy.^91^ Three participants wanted periodic communication about wait‐list position, prioritization rules and estimated procedure date. Online patient portals can improve patient experiences, behaviour and clinical outcomes by sharing personal information, engaging patients in their own care and promoting continuity of care.^92^ However, implementation and use of patient portals have been influenced by usability, patient characteristics and provider endorsement.^93^ Further research is needed to evaluate the uptake and impact of these interventions in the context of procedures delayed by COVID‐19.

This research identified several additional issues that warrant ongoing research. While wait times are a common health‐care challenge, few studies have assessed the impact on mental health, and even fewer evaluated strategies to support mental health. In particular, most of the included studies focussed on organ transplant; therefore, research is needed on how to support mental health among those waiting for a variety of procedures. Few studies assessed the influence of patient characteristics on mental health or the effectiveness of interventions, hence future research must consider explore these factors and generate insight on supports suitable for diverse individuals. The few studies involving caregivers, who were largely women, revealed they experience similar depression and greater anxiety compared with patients, so ongoing research could explore the attributes of supports beneficial to caregivers. With respect to the broader context, value‐based health care must be responsive to patient‐reported needs, preferences, experiences and outcomes. Given that this study identified relatively few studies that explored the mental health impact of waiting, future research might identify patient‐reported outcome measures related to the impact of waiting for procedures.

This study featured many strengths. We used rigorous methods,^18^, ^19^, ^21^, ^22^ searched multiple databases and complied with reporting standards for scoping reviews and search strategies.^20^, ^23^ By using a scoping review and including both quantitative and qualitative studies, we identified a discrepancy in interventions tested vs those desired by patients, thereby revealing patient‐centred strategies to employ in future. Also, by drawing on existing wait times literature, we identified strategies that may be relevant to the COVID‐19 context. Several limitations must also be noted. By restricting our search to English language studies, we may have omitted relevant studies published in other languages. The search strategy may not have identified all relevant studies, or our screening criteria may have been too stringent. Studies did not explicitly distinguish between anxiety and depression caused by waiting or by the underlying condition. Few studies reported sub‐analyses, so it is not fully apparent if findings apply to patients/caregivers who differ by socio‐demographic characteristics. Most studies focussed on transplant procedures, where patient anxiety stems from not knowing if they will live until an organ is available. Thus, the mental health impact may differ compared with waiting for other procedures with greater certainty of ultimately being treated. However, those undergoing pandemic‐imposed waits for time‐sensitive procedures with no clear resolution may experience similar mental health impact as those waiting for transplant.

## 5 CONCLUSION

This study emphasizes the need for policy and practice to implement strategies that support the mental health of wait‐listed patients and caregivers now and beyond COVID‐19. The need may be greater among women and new immigrants, and those of younger age, lower socio‐economic status, or with less‐positive coping ability or longer wait times. Patient‐centred strategies include a mechanism for affected persons to report mental health impact and hear that their concerns are acknowledged, support from peers to help them through the waiting period and periodic updates about position on the wait list and possible procedure date.

## CONFLICT OF INTEREST

None to declare.

## AUTHOR CONTRIBUTIONS

ARG, CYYY, JI, FCW, BR, HR, RG, SA, MPM and DES generated the idea. ARG, CYYY, JI, FCW, BR, HR, RG, SA, MPM and DES conceived and designed the experiments. ARG collected data. ARG, CYYY, JI, FCW, BR, HR, RG, SA, MPM and DES analysed data. ARG wrote the first draft of the manuscript. ARG, CYYY, JI, FCW, BR, HR, RG, SA, MPM and DES contributed to the writing of the manuscript. ARG, CYYY, JI, FCW, BR, HR, RG, SA, MPM and DES agreed with manuscript results and conclusions. ARG is the guarantor, had full access to the data in the study and takes responsibility for the integrity of the data and the accuracy of the data analysis.

## Supporting information

Table S1

Table S2

## Data Availability

All data are included in the manuscript and supplementary files.
