# Supplementary material for: The psychological burden of waiting for procedures and patient‐centred strategies that could support the mental health of wait‐listed patients and caregivers during the COVID‐19 pandemic: A scoping review
Source: Health Expect. 2021 Mar 26;24(3):978–90. doi: 10.1111/hex.13241 (PMC8235883; doi:10.1111/hex.13241)
Supplement: Supplementary file 1 — Table S1 [file HEX-24-978-s002.docx]

Supplementary Table 1. MEDLINE search strategy

| **#** | **Search Statement** | **Results** |
| --- | --- | --- |
| 1 | Waiting Lists/ | 11810 |
| 2 | (wait* adj4 list*).mp. | 21245 |
| 3 | (wait* adj4 time*).mp. | 14495 |
| 4 | wait-list.mp. | 2799 |
| 5 | waitlist*.mp. | 3056 |
| 6 | delay:.mp. | 546797 |
| 7 | Time-to-Treatment/ | 6371 |
| 8 | anxiety/ | 79956 |
| 9 | Psychological Distress/ | 423 |
| 10 | stress, psychological/ | 119341 |
| 11 | depression/ | 117414 |
| 12 | "Quality of Life"/ | 192255 |
| 13 | Physician-Patient Relations/ | 72145 |
| 14 | Trust/ | 9497 |
| 15 | Frustration/ | 2232 |
| 16 | Anger/ | 7637 |
| 17 | Fear/ | 31334 |
| 18 | Sadness/ | 107 |
| 19 | Obsessive behavior/ | 1213 |
| 20 | Behavior, addictive/ | 9736 |
| 21 | Substance-related disorders/ | 94776 |
| 22 | or/1-7 | 581692 |
| 23 | or/8-21 | 645546 |
| 24 | 22 and 23 | 15525 |
| 25 | limit 24 to (english language and yr="2010 -Current") | 8591 |
| 26 | limit 25 to (case reports or editorial or interview or lecture or letter or news) | 205 |
| 27 | 25 not 26 | 8386 |
