# Supplementary material for: The psychological burden of waiting for procedures and patient‐centred strategies that could support the mental health of wait‐listed patients and caregivers during the COVID‐19 pandemic: A scoping review
Source: Health Expect. 2021 Mar 26;24(3):978–90. doi: 10.1111/hex.13241 (PMC8235883; doi:10.1111/hex.13241)
Supplement: Supplementary file 2 — Table S2 [file HEX-24-978-s001.docx]

Supplementary Table 2. Data extracted from included studies (NR = not reported)

| Study  Year  Country  Key Finding | Goal  Disease  Procedure  Outcome | Research Design  Measures  Participants | Results | | |
| --- | --- | --- | --- | --- | --- |
|  |  |  | Impact of waiting on mental health | Determinants of mental health | Mental health intervention impact |
| Goktas^25^  2019  Turkey  Hope is influenced by perceived social support | Determinants of mental health impact of waiting  Kidney transplant  Hope | Survey  Herth Hope Scale, Multidimensional Scale  of Perceived Social Support  136 patients 18+ attending dialysis unit over 5 months; mean age 49, 44.1% female 67.6% married, 41.2% primary school grads  Race, ethnicity, culture NR | --- | Perceived social support (family) was positively associated with increasing age (H=17.2, p=0.002); hope was positively associated with being married (U=-2.3, p=0.023)  Perceived social support (family, friends) was positively associated with hope (r=0.276, p=0.001) | --- |
| Febrero^26,27^  2019, 2018  Spain  Group psychotherapy reduced depression | Intervention (group psychotherapy)  Liver transplant  Quality of life and depression | Before-after  Nottingham Health Profile  Beck’s Depression Test  7 wait-listed patients: 1 female, 6 males; aged 50 to 63; 2 primary, 3 secondary, and 2 higher education; 2 employed  A control group of 5 men with a mean age of 54 years were assessed for depression at the same two times  Race, ethnicity, culture NR | --- | --- | Quality of life improved but not significantly (2.8 before, 2.5 after, p=0.28)  All patients had several depressive symptoms before described as highs and lows within normality (range 0 to 10): sadness, disappointment, crying, anhedonia, lack of decisiveness, worse image, difficulty at work, difficulty sleeping, tiredness, lack of appetite, weight loss, worry about physical problems, loss of interest in sex. After, all patients scored 0. Depression in the control group did not improve at the second time. |
| Lopez Navas^28^  2019  Spain  Patients poorly adapt leading to psychological problems | Impact of waiting  Liver transplant  Psychological impact and coping | Survey  Mini Mental State Examination, International Neuropsychiatric Interview, Social Support Questionnaire, Mental Adjustment to Cancer Scale  112 wait-listed patients 18+; mean age 55; 22% female; 72% married; 25% had no education and 51% had minimal; none were actively working  Race, ethnicity, culture NR | 68% had symptoms of emotional psychopathology (depressive, anxious)  21% had non-functional family or social support; 92% were poorly adapted to the disease: 79% had a weaker fighting spirit, 51% expressed anxiety and concern about coping with the disease, 34% found they resorted to fatalism, 29% were in denial and 27% felt helpless | --- | --- |
| Lonning^29^  2018  Norway  HRQoL and mental health decreased in first year of waiting, more so for older patients | Impact of waiting and determinants  Kidney transplant  Health-related quality of life (HRQoL) | Survey at baseline, 6 and 12 months  Kidney Disease and Quality of Life Short form  261 patients aged 65+; mean age 71.1; 33% female; 69% on dialysis; 76.6% married  Race, ethnicity, culture NR | Mental component summary score decreased significantly (46.0 to 44.7, p<0.001)  Component scores were reduced for: Social function (72.3 to 51.9, p<0.001), Role limitations due to emotional problems (63.0 to 54.5, p=0.04), mental health (77.3 to 74.0, p=0.01) | Older age and being on dialysis were positively associated with low HRQoL (p<0.05) | --- |
| Agren^30^  2017  Sweden  Patients experience psychosocial impairment while waiting for transplant linked to coping style and younger age; caregivers experience burden | Impact of waiting and determinants  Heart/Lung transplant  HRQoL, anxiety, depression, stress, coping among patients, and burden for caregivers | Survey  EuroQol EQ-5D, Hospital Anxiety and Depression Scale, Impact of Event Scale, Mastery Scale, Caregiver Burden Scale  28 heart mean age 52 (+21 caregivers) and 26 lung mean age 50 (+19 caregivers) transplant patients aged 18+; 5 heart and 15 lung patients were female; 15 heart and 13 lung patients were married; 22 (55%) of caregivers were spouses; mean days on waiting list: 256 heart, 197 lung  Race, ethnicity, culture NR | Median health status: 50 heart and 39 lung  29% had doubtful anxiety  27% had doubtful depression  44% had medium to high intrusion stress and 38% had avoidance stress  Mean total coping score 19.3  22.9% of caregivers had medium to high burden levels | Lung patients had greater doubtful anxiety (p=0.04) and better coping (p=0.04) compared with heart patients; patients <50 years of age had greater coping (p=0.029) | --- |
| Annema^31^  2017  Netherlands  Patients experienced small to high levels of anxiety and depression linked to coping style | Impact of waiting and determinants  Liver transplant  Anxiety, depression, personal control, coping style | Survey  State-Trait Anxiety  Inventory short form, Center for Epidemiological Studies Depression scale, Mastery Scale, Coping Inventory for Stressful Situations  216 liver transplant patients aged 18+; mean age 51.6; 33% female; 87.8% with partner; 21.8% lower education; 29.6% employed; median time on waiting list 7.8 months (SD 13.9)  200 (92.6%) Dutch nationality but race, ethnicity, culture NR | 45.4% had anxiety above clinical levels  83.3% had depression above clinical levels  Mean personal control 23.9  Mean emotional coping 19.2  Mean task coping 25.1  Mean avoidance coping 17.1 | Experiencing more liver disease symptoms, low mastery, more use of emotional coping, and less use of task-oriented coping are associated with both anxiety (all p<0.001) and depression (all p<0.01) | --- |
| Bailey^32^  2017 United States  Telephone counseling and liver disease education similarly improved uncertainty, anxiety, depression and quality of life; telephone counseling increased self-efficacy | Intervention (phone call for uncertainty self-management)  Liver transplant patients and caregivers  Uncertainty, anxiety, depression, self-efficacy, quality of life | Randomized controlled trial  Patients:  Mishel Uncertainty in Illness Scale, Profile of Mood States, Center for Epidemiological Studies Depression Scale, Self-Control Schedule, Self-Efficacy Scale, Quality  of Life in Chronic Illness  Caregivers:  Perception of Uncertainty Scale-Family Member, Self-Control Schedule, Self-Efficacy Scale, Caregiver Reaction Scale  56 intervention dyads, 59 control dyads (liver disease education) aged 18+; mean age 55.4; female: 39% patients, 74% caregivers; 59% greater than high school education; 73.6% caregivers were spouse  52 (92.9%) of intervention dyads and 56 (94.9%) of control dyads were Caucasian | --- | --- | No differences were found between intervention and control group patients in change from baseline to 12 weeks for study outcomes except for self-efficacy (mean difference 3.1, 95% CI -4.4. to 10.7). Findings were similar for caregivers: self-efficacy mean difference 4.8 points, 95% CI -1.4 to 11.0 |
| Burns^33^  2017  Australia  Living with uncertainty and restricted activities were challenges | Impact of waiting  Kidney transplant  Lived experience | Qualitative (focus groups)  6 patients aged 29 to 63 with 10 to 72 months of dialysis experience  Race, ethnicity, culture NR | Living on dialysis is  physically and mentally demanding (transplant means freedom from dialysis, accepting wait as normal part of life)  Living with uncertainty (ongoing wait, uncertain outcome, fear of losing hope)  Altered relationship dynamics (limited ability to travel, work, and be social, valuing family support) | --- | --- |
| Carr^34^  2017  Canada  Coping mechanisms alleviated mental and emotional distress of living with uncertainty and restricted activities | Impact of waiting  Cardiac or orthopedic surgery  Effects of waiting | Qualitative (interviews)  32 patients; 10 cardiac, 22 orthopedic; 50% female; aged 43 to 89; median wait time: cardiac 6.5 days (range 3 to 60), orthopedic 102 days (range 41 to 218)  Race, ethnicity, culture NR | Restriction (pain, mobility, activities) and uncertainty (length of wait, future condition) caused mental and emotional distress  Resignation (passive acceptance, understanding high demand for service), coping (distraction, social support), and opportunity (use the time constructively to prepare for the procedure) lessened mental and emotional distress | --- | --- |
| Craig^35^  2017  Canada  Coping improved, which reduced anxiety and depression | Intervention (coping skills group therapy)  Kidney or liver transplant  Coping, anxiety, depression | Before-after  Brief COPE, Hamilton Anxiety Rating Scale, Hamilton Depression Rating Scale  41 adult patients; 21 female; median age 53 (range 19 to 65)  Race, ethnicity, culture NR | --- | --- | Significant improvement in some coping areas: decreased use of denial (M = 3.04 versus M = 2.46, n = 28, p<0.039) and self-blame (M = 4.27 versus M = 3.73 versus M = 3.30, n = 30, p<0.025), increased use of acceptance (M = 6.40 versus M = 7.07, n = 30, p<0.047), religion (M = 4.43 versus M = 5 .13, n = 30, p<0.042)  Anxiety (13.0 ± 1.23 versus 7.73 ± 0.85, <0.001) and depression (14.23 ± 1.45 versus  7.73 ± 0.95, p<0.0001) scores were significantly  reduced |
| Gross^36^  2017  United States  Mental HRQoL improved despite no impact on anxiety or depression | Intervention (phone mindfulness-based stress reduction)  Kidney transplant  Anxiety, depression, HRQoL | Randomized controlled trial  State-Trait Anxiety Inventory, Center for Epidemiologic Studies Depression Scale, SF-12 Physical and Mental Component Summaries  55 adult patients; mean age 54; 56% female; 49% married; 44% college graduate; 38% worked full-time; time on wait list 440.3 +/- 425.8 days; 27 intervention, 28 control (phone support group lacking mindfulness)  42 (76.0%) Caucasian, 8 (15.0%) Black, 6 (11.0%) Other | --- | --- | Changes in anxiety were small and did not differ between groups post-intervention (3.18 95% CI −1.47 to 7.82, p=0.18) or at 6-month follow-up (−1.88, 95% CI −8.14 to 4.37, p=0.55)  Depression improved in the control group post-intervention (2.81, 95% CI 0.02 to 5.60, p=0.05)  Mental HRQOL improved at follow-up for the intervention group (6.2 points, 95% CI 1.66 to 10.8, p= 0.01) |
| Hayes^37^  2017  United States  Increasing wait time was associated with increasing anxiety | Impact of waiting and determinants  Breast biopsy  Anxiety | Survey  State Anxiety Scale, Chronic Life Stressors Questionnaire, Traumatic Life Events Scale, Medical Outcomes Study Social Support Survey  140 women aged 21+; mean age 52; 58.6% married; 72.1% Caucasian; mean wait 6.15 days (SD 7.25, range 0 to 45)  101 (72.1%) Caucasian, 35 (25.0%) Black, 3 (2.1%) Asian, 1 (0.7%) American Indigenous | Mean anxiety 44.46 (SD 12.52, range 20 to 70); mean chronic life stress 15.86 (SD 10.99, range 0 to 46); mean traumatic life events 2.56 (SD 2.04, range 0 to 9), perceived social support 84.57 (SD 15.48, range 30 to 100) | Among women with low levels of chronic  life stress, increasing wait time was associated  with increasing anxiety (slope: B = 0.65, SE =  0.24, t = 2.69, p = .008). There was no relationship  between wait time and anxiety among  women with high levels of chronic life stress  (slope: B=−0.01, SE=0.12, t=−0.09, p=0.93). Anxiety was greater among Caucasian women (B=6.38, SE 2.30, p=0.006) | --- |
| Leong^38^  2017  Canada  Women waiting for pelvic organ prolapse surgery had similar emotional distress and mental impact as orthopedic patients | Impact of waiting  Pelvic organ prolapse, or hip or knee replacement among women  HRQoL | Survey  Short Form 36 Health Survey  250 women: 125 orthopedic, mean age 65; 125 pelvic, mean age 59; mean wait time: orthopedic 98 days +/- 120; pelvic 210 days +/-111, p<0.001  Race, ethnicity, culture NR | Physical health concepts were significantly worse for orthopedic patients (29.2 versus 41.5, p< 0.001)  Urogynaecology and orthopedic patients  had similar emotional and mental well-being (41.5 versus 44.6, p=0.09) | --- | --- |
| Miles^39^  2017  United Kingdom  Perceived delay caused cancer-related distress | Impact of waiting  Colorectal cancer treatment  Distress, quality of life, fear of cancer recurrence | Survey  Revised Impact of Events Scale, perceived diagnostic delay (yes/no), FACT-C, Fear of Cancer Recurrence Scale  296 patients; 50.7% female; 18.2% working; 98.3% Caucasian; mean age 69 (range 56 to 81); time since diagnosis ranged from 3.5 to 12 years  291 (98.3%) Caucasian | Distress – 6% scored 33 or higher indicating likely presence of PTSD; 19% reported poor quality of life; fear of recurrence was low mean 1.50 (SD 0.54, range 1 to 4)  26.7% perceived a diagnostic delay, which was correlated with higher cancer-related distress (0.052, 95% CI 0.001 to 0.103, p=0.045), mediated by quality of life, but not by fear of cancer recurrence | --- | --- |
| Nagao^40^  2017  Japan  Patients differed in anxiety based on coping style, not related to wait times | Impact of waiting and determinants  Endoscopic submucosal dissection for gastroenterological cancer  Coping, anxiety | Survey and qualitative interviews  General Self-Efficacy Scale  154 patients; 24.4% female; mean age 68.7 years (range 30 to 89); mean wait 46.28 days (SD 19.79, range 3 to 1717)  Race, ethnicity, culture NR | Self-efficacy: 14 “very low,” 39  “neutral,” 43 “high,” 3 “very high,” and 9 did not answer  Patients with no or little anxiety based on trust in doctor, passive acceptance, or optimistic outlook. Those with anxiety wanted the procedure as soon as possible to avoid cancer progression, metastasis, or recurrence, which led to feelings of impatience, depression, irritability and stress  Four waiting period coping types were identified:   1. Making phone inquiries 2. Busy and forgot about the medical procedure 3. Relief from anxiety 4. Unable to function well in daily life | There was no significant difference between self-efficacy, age, gender and wait times | --- |
| Sharman^41^  2017  Australia  Patients said mental health could be improved through peer support, counseling and communication about wait list position | Impact of waiting and suggestions  Bariatric surgery  Psychosocial impact | Qualitative (focus groups and interviews)  36 patients; 58.3% female; mean age 53 (range 23 to 66); mean wait 6 years (range 0 to 12)  Race, ethnicity, culture NR | Waiting was emotionally challenging (frustrating, depressing, stressful), led to weight gain and deteriorating  physical health (e.g. physical mobility) and psychological health (e.g. development of or worsening depression).  Even if wait times cannot be reduced, patients said the experience would be improved by: peer support, health and mental health counseling, and better communication about waitlist position and prioritization, even if wait times cannot be reduced | --- | --- |
| Burke^42^  2016  Australia  Education sessions did not improve distress or quality of life | Intervention (chronic pain education session)  Chronic pain care  Psychological distress, quality of life, pain acceptance | Randomized controlled trial  Kessler Distress Scale, World Health Organisation QOL-Brief Scale, Chronic Pain Acceptance Questionnaire  192 adults; mean age 44.1; 57% female; 38% married; 54% unemployed: 66 intervention, 126 control (wait list, no education session)  Location at birth: 258 (72.0%) Australia, 50 (14.0%) Europe, 17 (5.0%) Asia, 21 (9.0%) Other. Race, ethnicity, culture NR | --- | --- | The intervention did not impact distress (F=0.29, p=0.75), pain acceptance (F=0.73, p=0.48), or quality of life: physical (F=0.22, p=0.81), psychological (F=0.02, p=0.98) or social (F=0.61, p=0.54), or overall (F=0.20, p=0.82) |
| Fung-Zak Tsang^43^  2016  Canada  Patients were anxious, depressed and had impaired activities, and worried about progression; they did not need to develop coping strategies; instead they wanted a procedure date even if lengthy | Impact of waiting and suggestions  Endoscopic sinus surgery for chronic rhinosinusitis  Anxiety, depression, impairment | Multiple methods (survey, qualitative interviews)  Hospital Anxiety and Depression Scale, Work Productivity and Activity Impairment General Health Questionnaire  Questionnaire: 26 adults; mean age 50.7 +/-12.5 years; 46.2% female; mean wait time 216 days (range 31 to 425); Interviews: 15 adults, demographics NR  Race, ethnicity, culture NR | Mean anxiety/depression 14.7 +/- 8.6 (scores > 8 suggest high likelihood of clinically significant anxiety/depression  Impairment was 34.4% +/- 27.9 (range 0% to 90%)  Key themes:   - Patients were frustrated that wait was longer than expected - Fear of progression and being on wait list rather than their illness caused anxiety and depression - Most said they were coping and did not need to develop coping strategies - The only thing that would improve their experience was having a predetermined date regardless of length | --- | --- |
| Jin Chong^44^  2016  South Korea  While hopeful, they were tired of waiting; lacked insight on fairness of prioritization process | Impact of waiting  Kidney transplant  Psychosocial issues | Qualitative (interviews)  8 (5 men, 3 women) patients aged 39 to 67; on wait list mean 5.7 years (range 2 to 15); 5 married; 5 completed high school or college; 2 employed  Race, ethnicity, culture NR | Key themes:   - Last hope for a normal life - Constantly on alert for the phone call - Tired of waiting with no end in sight - Ambiguity about prioritization - Doubts about fairness - Grateful for family support - Mental preparation for a range of outcomes | --- | --- |
| Khatib^45^  2016  Australia  Distress linked with female, younger, and quality of life | Impact of waiting and determinants  Knee arthroplasty  Distress (depression, anxiety, stress) | Survey  Depression Anxiety and Stress Score, European Quality of Life Questionnaire, Knee Injury and Osteoarthritis Score  2,809 adults; 62% female; mean age 68 years  Race, ethnicity, culture NR | 26.8% reported distress (SD 0.44) | Those reporting distress were more likely to be female (p=0.025), younger (66.6 vs 68.8 years, p<0.001), significantly worse knee pain (28.9 vs 41.2, p<0.001), and general quality-of-health (p<0.001) | --- |
| Salci^46^  2016  Canada  Many patients feel sad or depressed | Impact of waiting  Anterior cruciate ligament surgery  Quality of life | Survey  ACL-QoL Questionnaire  50 patients; mean age 25.8 +/- 9.6 years; 42% female; mean wait 210.4 +/- 109.7 days  Race, ethnicity, culture NR | 51% of all patients reported feeling sad/depressed all or most of the time | --- | --- |
| Sutherland^47^  2016  Canada  Most patients had depression, which was higher younger and neurosurgery patients | Impact of waiting and determinants  Non-cancer elective surgery: general surgery, colorectal surgery, plastics, gynecology, otolaryngology, urology, orthopedics, and neurosurgery (total of 641 surgical procedures)  Anxiety, depression | Survey  EuroQoL’s EQ-5D (includes anxiety depression)  3,740 patients aged 19+ at 6 hospitals; mean age 56; 60% female  Race, ethnicity, culture NR | Overall mean depression score was 4.4 (SE 0.08); 76.3% reported some depression; 14.5% and 1.9% reported clinically significant and severe depression, respectively | Neurosurgery patients (p<0.01) and those aged <60 had higher depression (p<0.01) | --- |
| Teixeira^48^  2016  Brazil  Patients had high levels of anxiety not related to stress about mortality | Impact of waiting  Liver transplant  Anxiety, stress | Survey  State-Trait Anxiety Inventory, Perceived Stress Scale  52 adults; mean age 53.67 (SD 10.48); 21% female; 33% higher education; 75% married; 60% working; mean waiting time 571.85 (SD 534.62)  Race, ethnicity, culture NR | Mean anxiety 23.06 +/- 5.51 (98% had medium to severe anxiety)  Mean stress 12.10 +/- 5.62 points (8% had high stress)  Higher stress contributed to higher anxiety (r=0.4258, p<0.001) | --- | --- |
| Yngman-Uhlin^49^  2016  Sweden  Patients wanted to speak to staff about anxiety; wanted information on length of wait and process of prioritization | Impact of waiting  Kidney transplant  Waiting experience | Qualitative (interviews)  8 adults (1 woman) aged 33 to 53 years waiting at least 6 months (mean 34.75 months, range 12 to 72)  Race, ethnicity, culture NR | Key themes:   - Waiting was stressful and tiring, and grew harder over time - Patients felt tied up by treatment and by needing to be available for transplant - Wanted to be able to convey anxiety of staff - They expressed frustration about silence from the transplant unit and lack of information on how long they had to wait and how patients were prioritized | --- | --- |
| Matthews^50^  2015  Canada  Communication and empathy could alleviate dissatisfaction with waits | Impact of waiting  Breast, prostate, lung, or colorectal cancer patients  Satisfaction with waiting time | Qualitative (interviews)  60 adults; 45% female; 33.3% over age 65; 48.3% rural residence; 91.7% married; median wait time 840 days (range 0 to 897)  Race, ethnicity, culture NR | 3 inter-related factors were related to satisfaction:   - Interpersonal skill of treating physician (empathy, quality of information exchange - Coordination (scheduling, sharing information) - Timeliness of care (provider responsiveness to patient symptoms, shared sense of urgency between patient and providers) | --- | --- |
| Anthony^51^  2014  Canada  Anxiety, fear, depression and other psycho-social factors | Impact of waiting  Heart transplant (youth)  Psychosocial factors | Qualitative (interviews)  27 patients; 67% female; median age 15.5 years (range 12.2 to 18.4)  Race, ethnicity, culture NR | Key themes:   - Immense struggle across all aspects of life: physical, social, emotional, academic - Physical limitations/lethargy - Social isolation and limited social contacts - Anxiety, fear, depression | --- | --- |
| Brugger^52^  2014  Switzerland  Anxiety and depression common but coping style varied | Impact of waiting  Lung transplant  Emotions | Qualitative (interviews)  15 patients (7 women); mean age 52 years (range 27 to 65)  Race, ethnicity, culture NR | Most patients said they felt uncertainty, anxiety, depressed but varied in coping attitudes and behaviour  Patients develop different strategies to maintain positive identity and attitude, while preserving significant others from extra emotional load | --- | --- |
| dos Santos Cunha^53^  2014  Brazil  Waiting resulted in greater impact on mental health and depression among women | Impact of gender on mental health of wait-listed patients  Heart transplant  Psychological factors | Survey  SF-36 Quality of Life Questionnaire, Beck Depression Inventory  60 patients; 28.3% women; mean age 45.18 (SD 11.91, range 16 to 66); 68% elementary education; 58% employed; 73% married  Race, ethnicity, culture NR | --- | Women (mean 21.31, SD 12.82) were more depressed than men (mean 14.61, SD 9.43) but non-significant (p=0.06)  Women’s quality of  life was impaired in all domains compared to men, and was significantly poorer in emotional function (p=0.04) and mental  health (p=0.02) domains. | --- |
| Harrington^54^  2014  Canada  Women, new immigrants and longer wait time were more likely to have anxiety | Impact of waiting and determinants  Specialist visit for consultation or diagnosis for a new condition  Anxiety | Retrospective cohort  Canadian Community Health Survey data  2,516 patients; 59.6% female; 59.4% aged 45+; 64% living with partner; 69% Canadian born; 12% less than high school; 22.3% rural  69.1% Canadian-born, 24.0% immigrants >10+ years, 6.9% immigrants <10 years. Race, ethnicity, culture NR | Anxiety was the most frequently reported impact (69.1%) | Females (OR 0.74, p<0.05), those aged 30-59 (OR 1.49, p<0.05), and new immigrants (OR 1.95, p<0.05), those with longer wait times (OR 2.78, p<0.001) and those who perceived their wait as too long (OR 11.3, p<0.001) were more likely to report their life was affected | --- |
| Malik^55^  2014  Austria  Caregivers had greater anxiety than patients | Impact of waiting  Liver transplant  Distress, quality of life | Survey (baseline wait listing and 6 months)  Hospital Anxiety and Depression Scale, EORTC Quality of Life Questionnaire  47 patients; mean age 56.9 (SD 7.4); 21% female  24 caregivers; 84% were partners of the patient; mean age 50 (SD 14.2); 79% female  Race, ethnicity, culture NR | Quality of life among patients did not significantly change from baseline to 6 months  Caregivers anxiety was greater than patients at baseline (F=7.52, p=0.008) and 6 months (F=11.31, p=0.002), and increased over time  Caregivers and patients did not differ in depression scores at baseline or 6 months, and remained stable over time for both groups | --- | --- |
| Silva^56^  2014  Brazil  High anxiety accompanied by high stress | Impact of waiting  Kidney transplant  Anxiety, stress | Survey  Beck Anxiety Inventory, Lipp Stress Symptoms for Adults Inventory  50 patients; mean age 50.2 (SD 11.7); 54% female; time on transplant wait list mean 5.9 years (SD 4.4); 46% had a partner; 50% primary school education  Race, ethnicity, culture NR | 56% had anxiety (16% high); 60% had stress (30% severe), which was associated with longer wait list time (p=0.006)  Stress was 3.6 times (95% CI 1.34 to 9.89, p=0.011) more frequent among those with anxiety | --- | --- |
| Chin Ong^57^  2013  Singapore  Social function and mental health are impacted | Impact of waiting and determinants  Kidney transplant  HRQoL | Survey  Study Short Form 36-Item Health Survey  261 patients; mean age 47.8 (SD 8.0); 55.5% female; 44.2% secondary education; 72% married; 58.9% employed; 55% on dialysis 5+ years; wait list duration median 60 months (range 1 to 240)  207 (78.1%) Chinese, 42 (15.8%) Malay, 11 (4.2%) Indian, 5 (1.9%) Other | HRQoL scores were lower than general population-matched controls, and were clinically significant for social function (mean 75.9, SD 22.7) and mental component (mean 46.2, SD 10.6) summary scores  Wait time not significant | Being Chinese (physical B=-2.68; mental B=-2.62), married (physical B=-0.97; mental B=-4.35),  employed (physical B=-3.62; mental B=-2.97) and undergoing haemodialysis (physical B=-0.33; mental B=0.78) predicted better HRQoL scores (all p<0.05) | --- |
| Eskander^58^  2013  Canada  Anxiety and depression common, not associated with wait time | Impact of waiting  Thyroid surgery  Anxiety/depression | Survey  Perceived Stress Scale, Hospital Anxiety and Depression Scale  176 patients; mean age 53 (SD 12); 82% female; 75% married; 65% employed; 76% secondary education; mean wait for malignancy 107 days, for benign 218 days  176 (43.0%) born in North America. Race, ethnicity, culture NR | Mean stress score 22.8  Mean anxiety score 8.6 SD 4.62 (low range); mean depression score 11.8 SD 3.28 (moderate range); overall mean score 20.4 SD 4.69  Wait time not significant; stress (p=0.0003) and anxiety/depression (p=0.0157) decreased significantly after surgery | --- | --- |
| Gregory^59^  2013  Canada  Anxiety and frustration with healthcare system; lack of information; life on hold; perceived inequity; recommendations | Impact of waiting and suggestions  Bariatric surgery  Psychosocial impact | Qualitative (interviews)  27 patients; mean age 45.3 (range 26 to 64); 77.8% female; 66.7% married; 59.3% employed; 55.6% college education  Race, ethnicity, culture NR | Key themes:   - Perceived inequity in access (regional, socioeconomic) - Anger and frustration with healthcare system for not prioritizing bariatric surgery - Waiting was stressful, anxiety-provoking - Exasperated with the lack of information about position on the wait list - Confused about how wait list patients were prioritized for surgery - Uncertainty – lives on hold – unable to make future plans - Uncertainty reduced motivation to maintain healthy lifestyle - Recommendations to improve the experience were: periodic updates about position on wait list and reason for delay, and to alleviate concern about falling through the cracks/getting lost in the system; psychological counseling; access to a support group; patient mentors to help them through the waiting period | --- | --- |
| Yelle^60^  2013  United States  Depression; life on hold; trust in healthcare system eroded | Impact of waiting  Lung transplant  Psychosocial impact | Qualitative (interviews)  7 patients (3 women); demographics NR  Race, ethnicity, culture NR | Key themes:   - Depression - Unable to work - Life on hold/waiting by phone - Toll on relationships - Managing emotional roller-coaster daily is exhausting - Hurt/confusion of being lower priority erodes trust with provider | --- | --- |
| Dominguez-Cabello^61^  2012  Spain  Family anxiety or depression inversely related to patient coping | Impact of waiting  Liver transplant  Anxiety/depression among relatives, and coping with stress among patients | Survey  Hospital Anxiety and Depression Scale, Coping with Stress in Cancer  Patients Questionnaire  75 adult patient-relative dyads; demographics NR  Race, ethnicity, culture NR | Patients whose relatives had normal or moderate levels of anxiety/depression (versus clinical anxiety/depression) were more like to report coping with stress through seeking social support (p=0.007), emotional control (p=0.030), and active fighting (p=0.032) | --- | --- |
| Goetzinger^62^  2012  United States  Caregiver burden and coping style associated with depression and anxiety | Impact of waiting and determinants  Solid organ transplant  Depression, anxiety among caregivers | Survey  Beck Depression Inventory, State Trait Anxiety Inventory, Scale for Caregiver Burden, Medical Coping Modes Questionnaire  621 caregivers of 317 lung, 147 liver, 115 heart, 42 kidney patients; mean age 51.24 (SD 11.40); 78% female; 65% spouse; 65% employed  503 (82.0%) Caucasian, 81 (13.0%) African American, 37 (5.0%) Other | 17% had clinical depression (mean 7.32, SD 6.99) and 13% had clinical anxiety (mean 37.40, SD 11.18)  Mean burden score 8.21 (SD 6.44)  Mean coping scores:  Information seeking 14.32, SD 5.16; Social support 10.31, SD 3.30; Resignation 9.57, SD 2.94; Avoidance 4.18, SD 3.32 | Greater caregiver objective and emotional burden was associated with caregiver depression (b = 0.43, p < 0.001); greater caregiver objective burden (b = 0.38, p < 0.001) and avoidant coping style (b = 0.17, p = 0.002) were associated with anxiety | --- |
| Heilmann^63^  2012  Germany  Wait-listed patients more likely to use psychotherapy | Impact of waiting  Heart transplant  Psychosocial distress | Retrospective cohort  18 patients wait-listed 30+ days: mean age 50.7 (range 19 to 69); 16.7% female; and 20 who were not wait-listed for transplant: mean age 53.5 (range 28 to 73); 34.6% female  Race, ethnicity, culture NR | Wait-listed: 50% had an acute stress reaction, 11% had a depressive episode; not listed: 31% has an acute stress reaction, 15% had a depressive episode (p=0.202)  Wait-listed patients had more pre-transplant psychotherapy visits (mean 4, SD 7, range 0 to 27, median 2, IQR 5) than non-listed (mean 1, SD 1, range 0 to 4, median 0.5, IQR 1), p=0.011 | --- | --- |
| Kam-Tao Li^64^  2012  China  Female and longer wait associated with unhappiness | Impact of waiting and determinants  Solid organ transplant  Psychosocial impact | Survey  Developed a survey to assess perceived chance of getting transplanted, the level of happiness, and the perceived level of support received  442 patients waiting for kidney (93%), liver (4.1%, lung (1.8%), or heart transplant (1.1%); 79% aged 46+; 49% female; 63.3% waiting 3+ years  Race, ethnicity, culture NR | Issues of most concerns to the patients waiting for organ transplants were:  inconvenience of therapy (48.2%), disease progression (47.9%), burden to family (59.5%) and financial difficulties (52.3%); 21.7% of patients thought level of support from family and providers was inadequate. | Patients with longer transplant waiting times had lower self-estimated chance of receiving a transplant (p=0.004). Self-estimated chance of getting transplanted was positively associated with the happiness score (p<0.0001). More females (50.0% vs 25.7% in male) reported concerns about suffering  associated with the illnesses. | --- |
| Padwal^65^  2012  Canada  Waiting impacts quality of life | Impact of waiting and determinants  Bariatric surgery  Quality of life, satisfaction with care | Survey  Waiting List Impact Questionnaire  136 patients; mean age 43 (SD 9); 91% women; mean wait list 64 days (SD 76); mean visual analogue scale health status score 53/100 (SD 22)  138 (92.0%) Caucasian, 11 (7.0% Other | 47% agreed/strongly agreed that waiting affected their quality of life, 65% described wait times as concerning and 81% as frustrating; 31% were dissatisfied/very dissatisfied with overall medical care | Predictors of satisfaction were lower visual analogue scale health status scores (0.42, p=0.03), unemployment (13.7, p=0.01) and depression (10.3, p=0.003) | --- |
| Paul^66^  2012  Australia  Cancer patients experience concern about waiting across the treatment trajectory | Impact of waiting and determinants  Cancer diagnosis and treatment  Concern about wait by phase | Survey  Developed a survey to assess level of concern at several time points  146 patients; mean age 60, SD 14.1, range 19 to 90; 52% female; 83% urban; 79% high socioeconomic status; 84% 2 or less years since diagnosis; 34% breast, 17 prostate; 8.9% head and neck; 6.2% brain, 6.2% colorectal, 27% other  Country of birth: Australia 108 (74.0%), 38 (26.0%) other. Race, ethnicity, culture NR | 50% reported concern at one or more phases; 17% at all phases: Referral to specialist 25%, Referral to specialist visit 23%, Specialist visit to diagnosis 28%, Decision to date of surgery 23%, Decision to start of radiation (31%), Decision to start of chemotherapy (28%) | Those more likely to express concern were lower SES, born outside Australia, or younger age | --- |
| Santos^67^  2012  Brazil  Wait-listed patients reported depression and impaired quality of life | Impact of waiting  Liver transplant  Quality of life, depression | Survey  Short-Form 36 Quality of Life Questionnaire, Beck Depression Inventory  100 patients; mean age 47 (range 17 to 68); 27% female; 63% had partners; 34% primary school education; 27% employed  Race, ethnicity, culture NR | 64% had some level of depression, 10% moderate to severe; depression was associated with lower quality of life in all domains including social aspects (r=0.319, p=0.0012) and mental health (r=0.3832, p=0.001) | --- | --- |
| Ackerman^68^  2011  Australia  Waits cause high distress and declining quality of life | Impact of waiting  Hip or knee replacement  Quality of life, distress | Survey upon going on waiting list and before surgery  Assessment of Quality of Life scale, Kessler Psychological Distress Scale  134 patients; median age 67 (IQR 61 to 75); 59% female; 64% with a partner; 58% high school education; 70% retired; median wait 286 days, IQR 169 to 375  Race, ethnicity, culture NR | Overall HRQoL deteriorated over time (mean change -0.04, 95% CI -0.08 to -0.01, p=0.02)  Psychological distress was high at based and before surgery (mean change 0.5, 95% CI -0.4 to 1.4, p=0.25) | --- | --- |
| Jurado^69^  2011  Spain  Poor coping reduced quality of life | Impact of waiting  Liver transplant  Quality of life, coping | Survey  Short Form 36 Quality of Life Questionnaire, Medical Coping Modes Questionnaire  93 patients; mean age 53.92 (SD 9.1); 23.7% female; 36% primary school education  Race, ethnicity, culture NR | Non-active acceptance-resignation coping was negatively correlated with Emotional role (p=0.01) and social functioning (p=0.05) | --- | --- |
| Moran^70^  2011  Ireland  Geing on hold, uncertain future, losing hope | Impact of waiting  Kidney transplant  Experience of waiting | Qualitative (interviews)  16 adult patients; 43.8% female  Race, ethnicity, culture NR | Key themes:   - Living in hope for transplant - Uncertainty as time went on, unable to plan for future, hope turns to despair - Being on hold; unable to do many activities | --- | --- |
| Rodrigue^71^  2011  United States  Quality of life therapy improved quality of life and reduced distress | Intervention (quality of life therapy or supportive therapy)  Kidney transplant  Quality of life, distress | Randomized controlled trial  Quality of Life Inventory (QLI), Short Form 36 Health Survey mental health component score (SF36), Profile of Mood States-Short Form (POM), Hopkins Symptom Checklist-25 for anxiety, depression (HSC)  62 adults:  Quality of life therapy: 22 baseline, 20 at week 1, 17 at week 12; mean age 53.2 (SD 11.1); 46% female  Supportive therapy: 20 at baseline, 19 at week 1; 18 at week 12; mean age 48.6 (SD 11.9); 60% female  Control: 20 at baseline, 17 at week 1; 18 at week 12; mean age 52.7 (SD 12.7); 55% female  64%, 70% and 60% Caucasian, respectively | ---- | ---- | Quality of life therapy group had higher quality of life than supportive therapy or control at 1 (QLI: mean 46.7, SD 14.9, p<0.05; SF36: mean 46.2, SD 11.3) and 12 weeks (QLI: mean 45.8, SD 13.1, p<0.05; SF36: mean 46.1, SD 9.6, p<0.05))  Supportive therapy group had lower distress than control at 1 week (POM: mean 23.0, SD 22.7, p<0.05; HSC: mean 33.7, SD 15.6, p<0.05) but not 12 weeks  Quality of life therapy group had lower distress at 1 week compared with control (POM: mean 23.8, SD 18.1, p<0.05; HSC: mean 35.5, SD 13.7, p<0.05), and at 12 weeks (POM: mean 20.7, SD 16.1, p<0.05; HSC: mean 38.6, SD 8.3, p<0.05) compared with supportive therapy and control |
| Corruble^72^  2010  France  Anxiety and depression increased over time | Impact of waiting  Kidney transplant  Anxiety, depression | Survey upon listing, 12 and 24 months  State Trait Anxiety Inventory, Beck Depression Inventory  390 patients; mean age 46 (SD 12); 40.4% female; 68% unemployed  Race, ethnicity, culture NR | Anxiety (F=2.06, range 3 to 256, p=0.08) and depression (F=6.35, range 3 to 256, p=0.0004) progressively increased over time; over time, a greater proportion scored >13 for depression (8.7% to 16.7%, p<0.001), and greater than 40 for anxiety (42.1% to 55.4%, p<0.001) | --- | --- |
| Dominguez-Cabello^73^  2010  Spain  Relatives had higher anxiety than patients | Impact of waiting  Liver transplant  Anxiety, depression | Survey  Psychosocial Survey, Hospital Anxiety and Depression Scale  51 patient-relative dyads; patients: mean age 56.63 (SD 7.26), 13.7% female; relatives: mean age 49.29 (SD 11.49), 84.3% female; 68.6% were spouse  Race, ethnicity, culture NR | Relatives had higher anxiety (mean 10.80, SD 5.07, p=0.001) but similar depression (mean 6.65, SD 4.07, p=0.820) to patients | --- | --- |
| Mulcahy^74^  2010  Canada  Depressing, traumatizing, lack of information; mercy of health care system | Impact of waiting  Cancer diagnosis and treatment  Psychosocial experience | Qualitative (interviews)  22 patients and 4 family aged 19 to 71 years; 80.8% women; 38.5% married; 57.7% employed; cancers: breast, ovarian, colorectal, uterine, mouth, melanoma, and non-  Hodgkins lymphoma  Race, ethnicity, culture NR | Key themes:   - Frustrating, loss of control - Demoralizing - Traumatizing - Lack of information, at the mercy of the healthcare system - Depression | --- | --- |
| Parker^75^  2010  United Kingdom  Coping style, gender and waiting time influenced anxiety | Impact of waiting and determinants  Lower gastro-intestinal endoscopy  Anxiety, depression | Survey  Hospital Anxiety Depression Scale, Brief COPE  301 patients; demographics NR  Race, ethnicity, culture NR | Patients had greater moderate to severe anxiety than the general population (61% versus 12.6%, p<0.001)  Those with higher anxiety used coping strategies self-distraction, denial, behavioural disengagement, venting and self-blame | 56.3% of variance in anxiety was due to coping style (R^2^ 0.527 to 0.563 for different components), gender (R^2^ 0.121) and time on waiting list (R^2^ 0.058) | --- |
| Tiemi Miyazaki^76^  2010  Brazil  Caregivers have high levels of burden, stress and depression | Impact of waiting (caregivers)  Liver transplant  Perceived burden, depression | Survey  Caregiver Burden Scale, Beck  Depression Inventory  61 caregivers; mean age 47.6; 82% women; 64% spouses; 37.7% secondary schooling  Race, ethnicity, culture NR | Mean overall burden 1.69 points, SD 0.5694; mean depression 8.16 and 75.4% showing at least some depression  Key stressors were how to react in a crisis (42.6%) and patient mood swings (29.5%) and daily care (27.9%); 24.6% felt unprepared to perform their role: did not receive information of help from healthcare professionals; 59% had given up or reduced employment, negative effects on their social activities (41%), doubts about the future of the patient (65%), loss of privacy (29%), difficulty with concentration (75%), and insomnia (44%) | --- | -- |
